# Sedentary work and participation in leisure–time physical activity

**DOI:** 10.1007/s00420-021-01750-7

**Published:** 2021-09-15

**Authors:** Sven van As, Debby G. J. Beckers, Harm Veling, Wendela Hooftman, Michiel A. J. Kompier, Sabine A. E. Geurts

**Affiliations:** 1 Behavioural Science Institute, Radboud University, 6525 GD Nijmegen, The Netherlands; 2 TNO, Leiden, The Netherlands

**Keywords:** Leisure–time physical activity, Psychosocial job characteristics, Fatigue, Motivation, Occupational health

## Abstract

**Objective:**

Demanding psychosocial work characteristics, such as high job demands, can have a detrimental impact on leisure–time physical activity (LTPA), with adverse consequences for employee health and well-being. However, the mechanisms and moderators of this crossover effect are still largely unknown. We therefore aimed to identify and test potential mediating and moderating factors from within and outside the work environment. Based on the previous research, we expected job demands to be negatively related to LTPA through fatigue. In addition, we expected that job control and worktime control would attenuate the relationship between job demands and fatigue. Furthermore, we hypothesized that autonomous exercise motivation and spontaneous action planning would attenuate the relationship between fatigue and LTPA. In addition to these cross-sectional hypotheses, we expected the same effects to predict a change in LTPA in the following year.

**Methods:**

To investigate these assumptions, a preregistered longitudinal survey study was conducted among a large sample of Dutch employees in sedentary jobs. Participants reported on the constructs of interest in 2017 and 2018 (*N* = 1189 and 665 respectively) and the resulting data were analyzed using path analyses.

**Results:**

Our cross-sectional analyses confirm a weak indirect, negative association between job demands and LTPA, via fatigue. However, this finding was not observed in our longitudinal analyses and none of the other hypotheses were confirmed.

**Conclusion:**

This study shows that, among employees with relatively healthy psychosocial work characteristics (i.e., high job control), the evidence for an impact of these work characteristics on participation in LTPA is limited.

**Supplementary Information:**

The online version contains supplementary material available at 10.1007/s00420-021-01750-7.

## Introduction

Worldwide, more than a quarter of the adult population is insufficiently physically active (Guthold et al. 2018; Hallal et al. 2012). In high-income Western countries, levels of physical inactivity have increased in the last decades; from 31.6% in 2001 to 36.8% in 2016 (Guthold et al. 2018). This is alarming because regular physical activity is known to be a protective factor for health and well-being (World Health Organisation 2020). Therefore, it remains important to identify and understand the facilitators and barriers of physical activity.

Empirical evidence from longitudinal cohort studies, daily diary studies and experimental investigations suggests that high quantitative job demands (further referred to as job demands) such as time pressure and having much work to do, are associated with lower levels of physical activity during leisure time (Abdel Hadi et al. 2020; Fransson et al. 2012; Häusser et al. 2018; Häusser and Mojzisch 2017). Such findings are devastating because high job demands in itself already put employees at risk for developing ill-health and mental issues (Demerouti et al. 2001; Häusser et al. 2010; Karasek 1979) and leisure–time physical activity (LTPA) could act as a buffer against these negative consequences of demanding work (Sonnentag 2018). This indicates that especially employees who could benefit from physical activity find it difficult to be physically active during leisure time.

Exposure to high job demands is associated with elevated levels of fatigue, which is characterized by a reduced motivation to exert effort (Hockey 2011; van der Linden 2011). Especially for fatigued employees then, the expectation of high physical effort expenditure (Iodice et al. 2017) and the effortful processes that are required for initiating physical activities (cf. executive control; Kool et al. 2010) will be unappealing. Also, when performing physical activities, fatigued employees will be more likely to choose low-effort strategies (Hockey 2011) such as performing the exercise at a lower intensity or for a shorter duration. Thus, fatigued employees can be expected to be less physically active during leisure time as a result of a lower frequency, intensity or duration of physical activities.

Support for this reasoning comes from diary studies showing that fatigue negatively relates to LTPA (Niermann et al. 2016) and mediates the day-to-day negative association between job demands and LTPA (Häusser et al. 2018). Moreover, experimental studies have provided evidence for a negative causal impact of cognitively fatiguing work on subsequent physical behavior (for overviews, see Brown et al. 2020; van Cutsem et al. 2017). Together, these studies support the idea that fatigue explains the adverse pathway from high job demands to low LTPA in the short run (i.e., within days). Provided that job demands have both short-term and long-term accumulating effects on fatigue (Dicke et al. 2018; Ford et al. 2014; van der Linden 2011), these findings can be expected to also apply to long-term associations between job demands, fatigue and LTPA. However, the role of fatigue has not yet been investigated within the more stable negative association between job demands and LTPA.

Therefore, the first research question (***RQ1***) this large-scale study investigated was whether fatigue statistically accounted for the negative cross-sectional association between job demands and LTPA. In line with the previous studies, we hypothesized that job demands would be negatively related to LTPA (hypothesis 1; Fransson et al. 2012; Häusser et al. 2018) and positively to fatigue (hypothesis 2a; Häusser et al. 2018; Hockey 2011; van der Linden 2011). Moreover, we expected that fatigue would be negatively related to LTPA (hypothesis 2b; Ahola et al. 2012) and acted as an intermediary factor linking job demands to LTPA (hypothesis 2c; Häusser et al. 2018; Häusser and Mojzisch 2017).

### Work-related moderators

Multiple well-established theories suggest that job control buffers against the negative impact of high job demands on fatigue (Demerouti et al. 2001; Hockey 2011; Karasek 1979). Job control, also referred to as job autonomy, defines the extent to which employees can decide for themselves how to manage their job demands (Karasek 1979). Such control enables employees to flexibly switch to less demanding tasks when they feel the need to (Hockey 2011; Karasek 1979), which allows them to recover from demanding work already on the job (Meijman and Mulder 1998; Taris et al. 2006). This leaves employees less fatigued after work (Hockey 2011) and as such, job control could alleviate the negative impact of high job demands on fatigue (Hockey and Earle 2006).

The buffering effect of job control for fatigue can be expected to extend to LTPA as well. However, only indirect support for this buffering effect has been provided by previous research. Multiple studies found a positive association between job control and LTPA (for an overview, see Häusser and Mojzisch 2017) and the lowest levels of LTPA have been reported in jobs where demands are high while control is low (i.e., high-strain jobs; Fransson et al. 2012). Although these findings suggest positive consequences of job control for LTPA, the buffering effect of job control for LTPA has never been investigated in large-scale field research before.

An interesting form of job autonomy in this context is *worktime control* (WTC; Beckers et al. 2012), which entails the possibility for employees to control the duration and distribution of their working hours (Härmä 2006). Well-known examples are autonomy over starting and ending times of the working day and autonomy over the planning and length of breaks. Similar to general job control, WTC could buffer against the negative impact of job demands on work fatigue through recovery opportunities during the working day (i.e., recovery regulation; Nijp et al. 2012). By enabling employees to take well-timed breaks, employees can recover from demanding work already on the job (Meijman and Mulder 1998; Nijp et al. 2012), leaving them less fatigued afterwards. WTC could also directly enhance LTPA levels through improved time regulation (Nijp et al. 2012). Having control over start- and ending times of a working day enables employees to more flexibly combine working life and personal goals (e.g., physical activity). Despite this great potential of WTC for LTPA enhancement, WTC has never been studied in relation to LTPA before.

The second research question (***RQ2***) of this study therefore investigated to what extent job control and WTC buffered against the negative association between job demands, fatigue and LTPA. We hypothesized that job control and WTC would be positively related to LTPA (hypotheses 3a and 3b, respectively; Beckers et al. 2012; Häusser and Mojzisch 2017) and that they would attenuate the aversive association between job demands and fatigue (hypotheses 4a and 4b, respectively; Beckers et al. 2012; Hockey and Earle 2006).

### Person-related moderators

The third aim of this study was to investigate personal factors that could attenuate a negative association between work (fatigue) and LTPA. Two promising but as yet overlooked constructs in this context are autonomous motivation and action planning. *Autonomous motivation* is grounded in self-determination theory (SDT; Ryan and Deci 2000) and has repeatedly been linked to higher levels of sustained physical activity participation (Teixeira et al. 2012). Pursuing goals for autonomous reasons is experienced to be less effortful (Werner et al. 2016), which is relevant here because the willingness to exert effort tends to be lower when someone is fatigued (Hockey 2011; van der Linden 2011). As such, autonomous motivation for physical activity could alleviate the assumed negative association between fatigue and LTPA.

*Action planning* is a self-regulatory strategy in which individuals formulate a specific plan defining when, where and how the target behavior will be performed (Hagger and Luszczynska 2014). Given the observational nature of this study, we focus on spontaneous action plans here, which are self-set plans (Rise et al. 2003) and have been linked to elevated physical activity levels (Carraro and Gaudreau 2013). By making very specific plans for LTPA in advance, the cognitive effort that is required for planning physical activities is strategically shifted away from the moment someone is fatigued (Gollwitzer 1999; Gollwitzer and Sheeran 2006). This way, spontaneous action planning could attenuate the impact of fatigue on LTPA but this potential buffering effect has not been investigated in the work-context before.

Our third research question (***RQ3***) thus investigated to what extent autonomous exercise motivation and spontaneous action planning moderated the negative association between fatigue and LTPA. We expected that both autonomous exercise motivation and spontaneous action planning would attenuate the negative association between work fatigue and LTPA (hypotheses 5a and 5b, respectively; Gollwitzer and Sheeran 2006; Werner et al. 2016).

### Longitudinal associations

The final aim of this study was to investigate to what extent our predictors relate to LTPA *change* in the following year. Although such a longitudinal approach is crucial to advance insight into the directionality of the long-term associations between work and LTPA, only a very few studies tapped into this and with inconsistent results. Two studies among European employees did not provide evidence for an association between job demands and reductions in LTPA in the following year(s) (de Vries et al. 2016; Kouvonen et al. 2013) whereas a longitudinal study among Japanese employees did (Oshio et al. 2016). Regarding job control, two studies found (indirect) evidence for a negative association between job control and LTPA change in the following years (Fransson et al. 2012; Kouvonen et al. 2013). Finally, de Vries et al. (2016) found a negative relationship between work-related fatigue and physical activity change in the following year. Fatigued employees were more likely to reduce their levels of physical activity in the year thereafter. Thus, there is only limited and inconsistent evidence for the longitudinal associations between job demands, job control, fatigue and a subsequent change in LTPA.

Therefore, our fourth and final research question (***RQ4***) examined whether job characteristics (job demands, job control and WTC) were related to LTPA change in the following year. Based on our theoretical outline, we expected that high job demands would be related to a reduction in LTPA in the following year (hypothesis 6; Hockey 2011; van der Linden 2011). In addition, we expected that fatigue would be associated with a reduction in LTPA in the following year (hypothesis 7a; de Vries et al. 2016) and that the negative association between job demands and LTPA change would run via fatigue (hypothesis 7b). We specifically focused on fatigue at T1 here to tap into its long-term consequences for LTPA. Furthermore, we expected that job control and WTC positively related to LTPA change in the following year (hypotheses 8a and 8b, respectively; Beckers et al. 2012; Kouvonen et al. 2013). Finally, and in line with research question 4, we expected that autonomous motivation and spontaneous action planning at T2 buffered against the negative association between fatigue and LTPA change in the following year (hypotheses 9a and 9b, respectively; Gollwitzer 1999; Werner et al. 2016).

### The present study

**Fig. 1:**
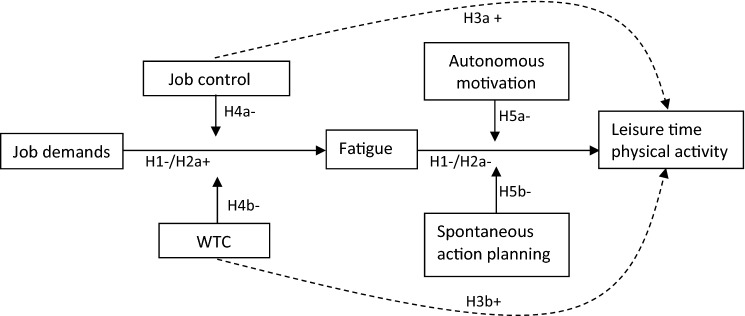
Path diagram visualizing the hypothesized cross-sectional associations (H). *Note.* ‘ + ’ indicates positive associations, ‘–’ indicates negative associations. The impact of job control and WTC (i.e., H3a and H3b versus H4a and H4b) will be investigated in separate analyses due to expected overlap in their effects

In summary, we aimed to advance our understanding of the impact of job demands on LTPA and took an innovative approach by combining insights from social–cognitive and occupational health psychology. From this broad perspective, we investigated (1) the mediating role of fatigue, (2) the moderating roles of the work-related factors job control and worktime control, (3) the moderating roles of the personal factors autonomous exercise motivation and spontaneous action planning and (4) the longitudinal effects of work on LTPA (see Fig. 1 for all cross-sectional hypotheses). To investigate these hypotheses, longitudinal survey data were collected among a large sample of Dutch sedentary workers. They reported on the constructs of interest twice, with a one-year time lag in between. Using path analyses, we investigated the assumed model as well as the cross-sectional and longitudinal pathways.

## Methods

### Participants

Participants were selected from the Netherlands Working Conditions Survey in 2016 (NWCS; Hooftman et al. 2017) which was conducted by the Netherlands Organization for Applied Scientific Research (TNO). This survey focused on the working conditions and employee health and well-being in the Netherlands. From an existing participant pool (*N* = 43,180), we approached employees who (1) worked for at least 32 h a week (high exposure to work demands), (2) sat behind their computer for at least 5 h per working day (i.e., sedentary work), (3) were not involved in physically demanding work and (4) did not have shift work (due to the potential consequences of a disrupted circadian rhythm and variance in leisure time for fatigue and LTPA; Atkinson et al. 2008), resulting in a total sample of 3000 approached employees*.* Of those, 1281 employees filled in the questionnaire in 2017 (cross-sectional dataset) and 772 employees filled in the questionnaire in both 2017 and 2018, providing full-panel data (longitudinal dataset). From these two datasets, participants were excluded because (1) they reported to work less than 32 h or (2) more than 48 h or (3) their age changed unrealistically (i.e., > 2-year increment or a decrement; only applies to longitudinal data), resulting in two final samples consisting of 1189 employees (cross-sectional, 39.63% of the approached sample) and 656 employees (full-panel longitudinal, 21.87% of the approached sample; See Table A1 for details). As can be seen in Table 1, the investigated samples consisted of relatively more men, were relatively well educated and worked on average more hours per week than the original sample.

**Table 1:** Means, standard deviations and frequencies of demographic variables, per subsample

	Original (*N* = 43,180)	Cross-sectional (*N* = 1189)	Longitudinal (*N* = 656)
	*M* (*SD*)/%	*M* (*SD*)/%	*M* (*SD*)/%
Gender	52.5% male	66.4% male	68.3% male
Age	42.2 (14.1)	44.83 (11.32)	46.23 (10.81)
Education	Low = 18.9% Intermediate = 42.8% High = 38.3%	Low = 4.7% Intermediate = 32.6% High = 62.7%	Low = 3.8% Intermediate = 31.9% High = 64.3%
Working hours	29.4 (11.9)	37.53 (2.94)	37.39 (2.97)

### Measures

#### Job demands

Three items of the subscale ‘psychological demands’ from the Dutch version of the Job Content Questionnaire (Houtman 1995; Karasek et al. 1998) were used to measure job demands (e.g., ‘Do you have to work fast?’; 1 = *Never*, 4 = *Always*). A high score on this scale represents high levels of job demands. Internal consistency of the scale was good in both waves (Cronbach’s α = 0.81 in 2017 and 0.82 in 2018).

#### Job control

Three items of the ‘control’ subscale from the Dutch version of the Job Content Questionnaire (Houtman 1995; Karasek et al. 1998) measured job control (e.g., ‘Do you decide for yourself how to do your work?’; 1 = *no*, 2 = *yes sometimes,* 3 = *yes, regularly*). A high score on this scale represents high task control. Internal consistencies were acceptable in both waves (Cronbach’s α = 0.77 in 2017 and 0.74 in 2018).

#### Worktime control

Four questions from the WTC-access subscale (Nijp et al. 2015) were selected to measure worktime control (e.g., ‘To what extent do you have the possibility to determine yourself when to take a break’ and ‘To what extent do you have the possibility to determine the starting and ending times of your working day yourself’; 1 = *(Almost) not at all,* 5 = *To a very high extent*). A high score on this scale indicates high levels of worktime control and internal consistencies of this scale were good in both years (Cronbach’s α = 0.84 in 2017 and 0.84 in 2018).

#### Fatigue

We adapted the Fatigue Assessment Scale (FAS; Michielsen et al. 2004) to measure work fatigue *after workdays*. The scale consisted of 7 items tapping into fatigue after work (e.g., ‘After a working day, I have enough energy for everyday life’; 1 = *never*, 5 = *always*). A high score on this scale represents high levels of work fatigue. The scale is a valid and reliable measure of work fatigue among the working population (de Vries et al. 2003) and showed good internal consistencies (Cronbach’s α = 0.88 in 2017 and 0.89 in 2018).

#### Spontaneous action planning

A Dutch translation of the original four-item scale (Sniehotta et al. 2005) was used to assess the extent to which individuals make detailed plans regarding when, where and how to perform their physical exercises (e.g., ‘Each week, I plan when to exercise’, 1 = *completely disagree*, 4 = *totally agree*). A high score on this scale indicates high levels of spontaneous action planning. The scale showed excellent internal consistencies (Cronbach’s α = 0.97 in 2017 and 0.96 in 2018).

#### Autonomous exercise motivation

The total score of the subscales ‘identified regulation’, ‘integrated regulation’ and ‘intrinsic regulation’ from the Behavior Regulation in Exercise Questionnaire (BREQ-4; Markland 2017) was used to measure autonomous exercise motivation (e.g., ‘I exercise because the benefits are important to me’ (identified),’I exercise because it is part of who I am’ (integrated), ‘I exercise because it’s fun’ (intrinsic); 1 = *does not apply to me*, 7 = *strongly applies to me*). A high score on this scale indicates high levels of autonomous exercise motivation. The scale showed excellent internal consistencies (Cronbach’s α = 0.96 in 2017 and 0.96 in 2018).

#### Leisure–time physical activity

The Dutch version of the ‘leisure time’ subscale from the International Physical Activity Questionnaire (IPAQ, long version, last 7 days; Craig et al. 2003; van Poppel et al. 2004) was used to measure frequency, duration and intensity of LTPA. Specifically, participants reported the number of days per week as well as the time per day spent on walking, moderate intensity activities and vigorous intensity activities. Each activity has its own MET score, which is the metabolic equivalent of a task, expressed as milliliters oxygen per kilogram bodyweight per minute (Ainsworth et al. 2011). A MET score of 1 represents energy expenditure during rest and is approximately 3.5 ml O_2_ kg^−1^ min ^−1^ in adults. Following the IPAQ scoring protocol, walking, moderate intensity and vigorous intensity activities had MET scores of 3.3, 4.0 and 8.0 respectively. Weighted weekly activity scores were then calculated as duration × frequency × MET score and expressed as MET hours per week (MET^.^hours^.^wk^−1^). This score thus represents the frequency, duration as well as intensity of physical activity during leisure time. The IPAQ has been tested internationally as well as in the Netherlands, and is a reliable and reasonably valid questionnaire for physical activity assessment (Craig et al. 2003; van Poppel et al. 2004).

#### Control variables

As preregistered on the Open Science Framework, we selected the five variables that correlated most strongly to the dependent variable (LTPA1 for cross-sectional, LTPA2 for longitudinal) as control variables in the analysis (for an overview of all variables included in this correlation analysis, see Supplementary Table S1 and https://osf.io/g7xb3/): *Controlled exercise motivation* was measured with 12 items from the subscales ‘external regulation’, ‘introjected avoidance regulation’ and ‘introjected approach regulation’ of the BREQ-4 (Markland 2017; e.g., ‘I exercise because other people say I should’; 1 = *does not apply to me*, 7 = *strongly applies to me*). A high score on this scale represents high levels of controlled exercise motivation. Internal consistencies of the scale were good (Cronbach’s α = 0.862 in 2017 and 0.865 in 2018). *Amotivation* was measured with four items of the ‘amotivation’ subscale of the BREQ-4 (Markland 2017; e.g., ‘I think exercising is a waste of time’; 1 = *does not apply to me*, 7 = *strongly applies to me*). Higher scores represent higher levels of amotivation. Again, internal consistencies of the scale were good (Cronbach’s α = 0.836 in 2017 and 0.898 in 2018). *Physical activity in other domains* was measured with the slightly adapted subscales ‘commuting activities’, ‘activity at work’ and ‘household activities’ of the Short QUestionnaire to Assess Health Enhancing Physical Activities (SQUASH; Wendel-Vos et al. 2003). For each domain, participants reported the number of days per week and the time per day spent on light/moderate or vigorous activity. In addition, pace of cycling/walking was reported in the commuting subscale. Each type of activity again had its own MET score and a weighted activity score was calculated as frequency x duration x MET score per domain. These domain scores were summed to represent total MET minutes per week spent on activities in alternative domains (for a detailed description, see Wendel-Vos et al. 2003). *Composition of physical activities* was measured with the single-item measure ‘Do you perform your leisure–time physical activities together or alone?’ (coded as 1 = *always alone*, 2 = *sometimes together*). *Timing of physical* activities was measured with the question ‘Do you determine for yourself when to be physically active?’ (coded as 1 = *no (e.g., scheduled training every week)*, 2 = *sometimes,* 3 = *yes (e.g., you train for yourself*)). Finally, *dog ownership* was measured with a single-item measure ‘Do you own a dog?’ (1 = *no*, 2 = *yes*).

### Statistical approach

Prior to analyzing, descriptive statistics were obtained to gain insight into the general distributions of the central constructs. Also, correlations among the core constructs of interest were obtained to investigate the patterns of relationships and to identify the control variables to be included in the main analyses. Variables that were part of interaction terms were centered before creating the interaction terms (i.e., job demands, job control, WTC, fatigue, autonomous motivation, spontaneous action planning). Next, patterns of missingness were inspected to determine whether multiple imputations were required to prevent biases (Newman 2009).

#### Confirmatory analyses

To investigate our research questions and in line with our preregistration (see https://osf.io/g7xb3/), two path analyses were conducted to investigate our hypotheses.^1^ To conduct these analyses, four models were fitted with the ‘sem’ function of the lavaan package (Rosseel 2012) in the statistical R-environment (R Core Team 2020). All parameters were estimated so that the models were saturated (i.e., *df* = 0), however, only hypothesized pathways will be reported here for convenience (see https://osf.io/g7xb3/ for exact specifications). A robust estimator was implemented to deal with non-normality of variables and multiple imputation was performed to prevent biases from nonrandom missingness (Newman 2009).

For answering research questions 1 till 4, the conceptual model was tested cross-sectionally twice, with either job control or WTC as moderator.^2^ In line with our preregistration, we controlled for physical activities in other domains, dog ownership, controlled motivation, amotivation and social composition of LTPA within the cross-sectional analyses. For research question 5, a similar approach was taken in two longitudinal analyses (including either job control or WTC at T1 as moderator) with only a few deviations. LTPA at T2 (2018) instead of LTPA at T1 (2017) served as the outcome measure while LTPA1 was included as control variable. Thereby we tested the associations of job demands, job control and work fatigue with LTPA-*change* over the one-year time interval. In addition, the T2 measures of autonomous motivation, spontaneous action planning and control variables were included in the models instead of T1 measures. Finally, we controlled for the timing of LTPA, controlled motivation, amotivation and dog ownership within the longitudinal analyses.

#### Robustness analyses

Three types of robustness analyses were performed. To investigate whether findings were driven by influential cases, the models were re-tested while excluding cases with outliers (i.e., SD > 3) on the respective dependent variable (i.e., LTPA T1 or T2). Second, we re-tested the longitudinal models while excluding all participants that had dropped out at T2 (i.e., only testing participants providing full-panel data) to investigate whether in- or exclusion of these participants mattered for the results (robustness check). Third, all models were tested while excluding all of the original control variables to investigate whether inclusion of these variables affected the results.

## Results

Table 2 provides an overview of the core constructs of interest and their inter-correlations. As can be seen in the table, none of the psychosocial work characteristics in year one (i.e., job demands, job control and WTC) correlated to LTPA in year one or year two (*p*’s > 0.05). However, they did correlate to work fatigue in the expected directions, which in turn correlated to LTPA in both years. Further inspection of our data reveals that only 15.1% of the employees in our cross-sectional sample was insufficiently active in 2017 (i.e., less than 7.5 MET hours per week). With regard to our full-panel data, only 9.2% was insufficiently active in 2017 and only 8.5% in 2018. This is much lower than the 42.2% of the 18–65 years old Dutch population who report to be insufficiently active at moderate intensity (StatLine 2020; for an overview of all other variables, see Table A2).

**Table 2:** Means, standard deviations and correlations of main variables (*N* = 656)

Variable	*M/ %*	*SD*	Theoretical range	1	2	3	4	5	6	7	8	9	10	11	12
1. Age	45.27	10.80	18–∞												
2. Gender	0) 68.3% 1) 31.7%		0 = male 1 = female	− 0.11**											
3. BMI	25.86	4.36	∞	0.23**	− 0.02										
4. LTPA T1^a^	28.72	25.25	∞	− 0.05	− 0.03	− 0.10*									
5. LTPA T2^a^	30.16	28.28	∞	0.09*	− 0.06	− 0.08	0.47**								
6. Fatigue T1	2.25	0.65	1–5	− 0.05	0.05	0.16**	− 0.15**	− 0.14**							
7. Fatigue T2	2.27	0.67	1–5	− 0.08*	0.07	0.13**	− 0.13**	− 0.12**	0.71**						
8. Job demands T1	2.41	0.55	1–4	0.02	0.07	0.05	0.02	− 0.03	0.32**	0.27**					
9. Job demands T2	2.39	0.56	1–4	− 0.06	0.06	− 0.02	0.04	0.01	0.21**	0.31**	0.60**				
10. Job control T1	2.68	0.44	1–3	0.11**	− 0.03	− 0.10*	− 0.04	0.04	− 0.26**	− 0.21**	− 0.23**	− 0.11**			
11. Job control T2	2.72	0.41	1–3	0.12**	− 0.00	− 0.04	− 0.04	0.02	− 0.24**	− 0.29**	− 0.16**	− 0.20**	0.54**		
12. WTC T1	3.34	1.03	1–5	− 0.01	0.00	− 0.05	0.01	0.07	− 0.14**	− 0.21**	− 0.04	− 0.01	0.44**	0.41**	
13. WTC T2	3.39	1.03	1–5	− 0.04	0.01	− 0.06	0.01	0.04	− 0.19**	− 0.25**	− 0.08*	− 0.05	0.36**	0.48**	0.74**

^a^MET hours per week

*p < 0.05. **p < 0.01

### Confirmatory analyses

#### Cross-sectional analyses

An overview of all relevant estimates^3^ of the cross-sectional analyses (research questions 1–3) is presented in Table 3. As the overlapping pathways of the two cross-sectional analyses were almost identical, we only report the statistics of the analysis with job control as moderator here. The results show that the total association between job demands and LTPA (i.e., including the indirect pathway via fatigue) was not significant (*p* = 0.122). However, when looking at the specific pathways, job demands were weakly related to LTPA (*β* = 0.073, *p* = 0.016; hypothesis 1), moderately to fatigue (*β* = 0.240, *p* < 0.001; hypothesis 2a) and that fatigue was negatively and weakly related to LTPA (*β* =  − 0.115, *p* < 0.001; hypothesis 2b). Importantly, the indirect pathway from job demands, through work fatigue, to LTPA was significant and in the expected, negative direction (*β* =  − 0.028, *p* = 0.001; hypothesis 2c). Thus, our findings with respect to research question 1 partly confirm our hypotheses: Job demands are weakly and negatively related to LTPA through fatigue. However, the weak direct positive relation between job demands and LTPA was unexpected.

**Table 3:** Estimates and significance levels of the hypothesized cross-sectional pathways (*N* = 1189)

Pathway	Model 1 (Job Control)			Model 2 (WTC)		
	*z*	*β*	*p*	*z*	*β*	*p*
Outcome: LTPA						
Job demands	2.400	0.073	0.016	2.811	0.085	0.005
Job control	− 1.868	− 0.075	0.062	−	−	−
WTC	−	−	−	− 2.937	− 0.089	0.003
Work fatigue	− 3.823	− 0.115	0.000	− 3.578	− 0.108	0.000
Autonomous motivation	5.370	0.204	0.000	5.716	0.213	0.000
Action planning	5.315	0.154	0.000	5.400	0.158	0.000
Autonomous motivation × Work fatigue	− 2.449	− 0.070	0.014	− 2.600	− 0.073	0.009
Action planning × Work fatigue	0.562	0.017	0.574	0.644	0.019	0.520
Outcome: Work fatigue						
Job demands	8.065	0.240	0.000	9.466	0.281	0.000
Job control	− 5.672	− 0.200	0.000	−	−	−
WTC	−	−	−	− 4.207	− 0.124	0.000
Job demands × Job control	− 0.781	− 0.028	0.435	−	−	−
Job demands × WTC	−	−	−	1.367	− 0.042	0.172
Indirect pathway						
Job demands → Work fatigue → LTPA	− 3.453	− 0.028	0.001	− 3.342	− 0.030	0.007
Job control → Work fatigue → LTPA	3.115	0.023	0.002	−	−	−
WTC → Work fatigue → LTPA	−	−	−	2.694	0.013	0.007
Total pathway						
Job demands → LTPA	1.548	0.045	0.122	1.907	0.055	0.057
Job control → LTPA	− 1.301	− 0.052	0.193	−	−	−
WTC → LTPA	−	−	−	− 2.524	− 0.076	0.012

*Note*. In both models, we controlled for alternative PA, dog ownership, controlled motivation, amotivation and PA group composition. *Z* values represent the Wald statistics and *β*’s are the standardized parameter values obtained from the completely standardized solution

Concerning research question 2, job control was not significantly related to LTPA (*p* = 0.062; hypothesis 3a) and did not moderate the relationship between job demands and work fatigue (*p* = 0.435; hypothesis 4a). Thus, in contrast to our expectations, we found no evidence for job control to be positively related to LTPA or buffer against an aversive relationship between job demands, fatigue and LTPA. Furthermore, we found that WTC was weakly *negatively* related to LTPA (*β* = − 0.089, *p* = 0.003; hypothesis 3b) and we did not find evidence for WTC to moderate the association between job demands and work fatigue (*p* = 0.172; hypothesis 4b). Thus, employees who reported more WTC had slightly lower levels of LTPA and WTC did not attenuate an aversive relationship between job demands, fatigue and LTPA.

**Fig. 2:**
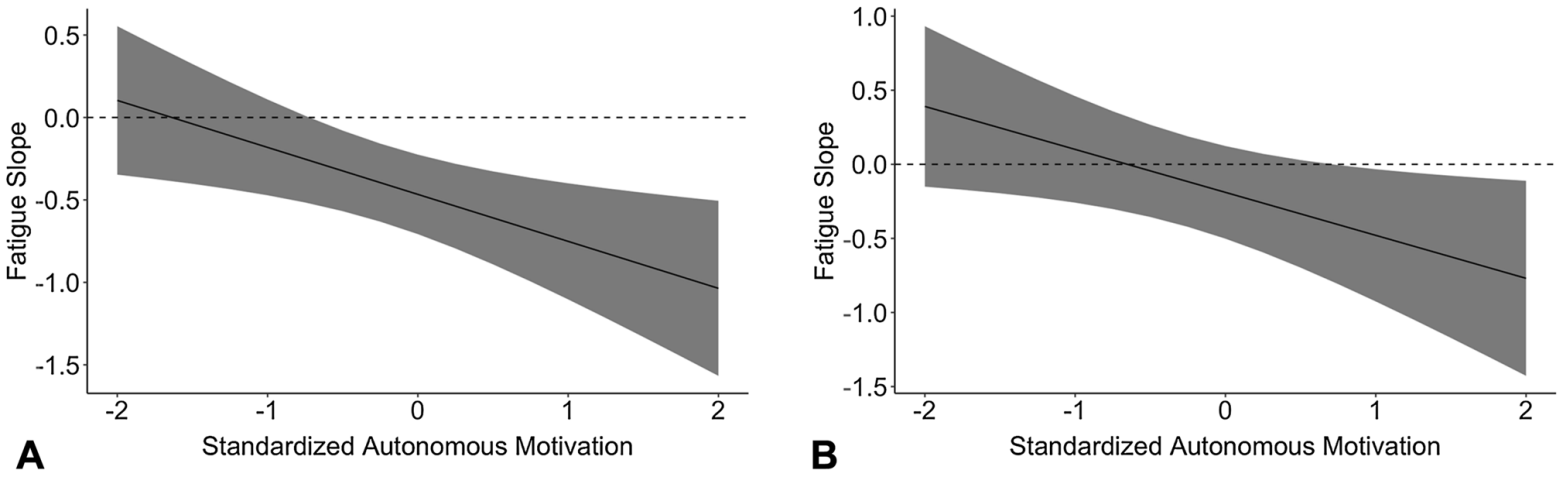
The two-way interaction between fatigue and autonomous motivation in the cross-sectional (**A**) and longitudinal (**B**) analyses. *Note.* The solid black lines represent the fatigue slope at each level of standardized autonomous motivation. Shaded areas represent the 95% confidence intervals of the fatigue slope estimates. Absence of overlap between the shaded area and dashed 0-line indicate significance of the fatigue slope

Our results for research question 3 showed that autonomous motivation moderated the relationship between fatigue and LTPA (*p* = 0.014; hypothesis 5a). However, the simple slope analysis indicated that the negative association between work fatigue and LTPA was *strengthened* by autonomous motivation (see Fig. 2a). Thus, in contrast with our expectation, the association between fatigue and LTPA (i.e., the fatigue slope) became slightly more negative when levels of autonomous motivation increased. Finally, spontaneous action planning did not moderate the association between work fatigue and LTPA (*p* = 0.574; hypothesis 5b).

#### Longitudinal analyses

Table 4 shows all estimates and *p* values of the longitudinal analyses investigating research question 4. As can be seen, the total association between job demands at T1 and LTPA *change* (i.e., also including the indirect effect via fatigue) was not significant (*p* = 0.383). Also, when looking at the specific pathways, job demands at T1 did not predict LTPA change in the following year (*p* = 0.570; hypothesis 6) and neither did fatigue at T1 (*p* = 0.273, hypothesis 7a). In line with these findings, also the indirect pathway from job demands at T1, through work fatigue at T1, to LTPA change was not significant (*p* = 0.277; hypothesis 7b). Thus, in contrast to our expectations, job demands and fatigue did not (indirectly) predict LTPA change in the following year.

**Table 4:** Estimates and significance levels of the hypothesized longitudinal pathways (*N* = 1189)

Pathway	Model 1 (Job Control)			Model 2 (WTC)		
	*z*	*β*	*p*	*z*	*β*	*p*
Outcome: LTPA change						
Job demands T1	− 0.569	− 0.021	0.570	− 0.690	− 0.025	0.490
Job control T1	1.023	0.037	0.306	−	−	−
WTC T1	−	−	−	0.871	0.035	0.384
Work fatigue T1	− 1.095	− 0.041	0.273	− 1.150	− 0.044	0.250
Autonomous motivation T2	2.706	0.146	0.006	2.785	0.149	0.005
Action planning T2	1.824	0.082	0.068	1.782	0.081	0.075
Autonomous motivation T2 × Work fatigue T1	− 2.163	− 0.078	0.031	− 2.248	− 0.081	0.025
Action planning T2 × Work fatigue T1	0.219	0.008	0.827	0.305	0.012	0.760
Outcome: Work fatigue T1						
Job demands T1	8.717	0.258	0.000	10.109	0.302	0.000
Job control T1	− 6.150	− 0.220	0.000	−	−	−
WTC T1	−	−	−	− 4.807	− 0.139	0.000
Job demands T1 × Job control T1	− 0.886	− 0.032	0.375	−	−	−
Job demands T1 × WTC T1	−	–	−	− 1.240	− 0.039	0.215
Indirect pathway						
Job demands T1 → Work fatigue T1 → LTPA Change	− 1.087	− 0.011	0.277	− 1.141	− 0.013	0.254
Job control T1 → Work fatigue T1 → LTPA Change	1.075	0.009	0.283	−	−	−
WTC → Work fatigue T1→ LTPA Change	−	−	−	1.129	0.006	0.259
Total pathway						
Job demands T1 → LTPA Change	− 0.872	− 0.031	0.383	− 1.064	− 0.038	0.287
Job control T1 → LTPA Change	1.197	0.044	0.231	−	−	−
WTC T1 → LTPA Change	−	−	−	1.016	0.041	0.309

*Note*. In both models, we controlled for alternative PA, dog ownership, controlled motivation, amotivation and PA group composition. *Z* values represent the Wald statistics and *β*’s are the standardized parameter values obtained from the completely standardized solution

Concerning the potential buffering factors, job control at T1 did not significantly predict LTPA change (*p* = 0.306; hypothesis 8a) and neither did WTC at T1 (*p* = 0.384; hypothesis 8b). However, autonomous motivation at T2 moderated the relationship between work fatigue at T1 and LTPA change (*p* = 0.031; hypothesis 9a). As can be seen in Fig. 2b, the association between fatigue at T1 and LTPA change became slightly negative when autonomous motivation was high. This indicates that, in contrast to our expectation, autonomous motivation strengthened a weak negative association between fatigue and LTPA change. Finally, spontaneous action planning at T2 did not moderate the association between work fatigue at T1 and LTPA change (*p* = 0.827; hypothesis 9b). Thus, in contrast to our expectations, job control and WTC did not predict an increase in LTPA. Moreover, autonomous motivation and spontaneous action planning did not buffer against a negative association between fatigue LTPA change in the next year and autonomous motivation even seemed to strengthen it.

### Robustness analyses

Three sets of analyses were performed to test the robustness of our findings. In the first set of robustness analyses, cases with outliers (i.e., > 3SD) in LTPA were excluded (*n* = 19 for cross-sectional data and *n* = 16 for longitudinal data). In the robust cross-sectional analysis with job control as moderator, job demands were not significantly related to LTPA anymore (*p* = 0.085; hypothesis 1). In the longitudinal analysis with job control as moderator, the Fatigue × Autonomous Motivation interaction became insignificant (*p* = 0.057; hypothesis 9a). In the robust longitudinal analysis with WTC as moderator, no significant changes occurred with respect to our hypotheses when outliers in LTPA at T2 were excluded (see Table A3 for an overview). These findings show that most of our findings were robust but that the unexpected positive relationship between job demands and LTPA as well as the longitudinal moderating effect of autonomous motivation may have been driven by outliers instead of representing a true effect for the entire sample.

In the second set of robustness analyses, we excluded participants from the longitudinal analyses who did not provide T2 data (i.e., only including full-panel participants). Exclusion of these participants did not affect the original findings. In the third set of robustness analyses, we did not control for any variables anymore. Here too, no changes with respect to our hypotheses were observed. Thus, in- or exclusion of (1) participants who dropped out or (2) control variables did not determine our findings. Table 5 provides an overview of evidence for all hypotheses tested in the main and robust analyses.

**Table 5:** Synthesis of Evidence for all Hypotheses

Research questions and hypotheses	Evidence		
	Main	Robust 1^a^	Robust 2^b^
Research question 1			
* Hypothesis 1: Job demands negatively relate to LTPA*	X!	X/X!	X!
* Hypothesis 2a: Job demands negatively relate to fatigue*	√	√	√
* Hypothesis 2b: Fatigue negatively relates to LTPA*	√	√	√
* Hypothesis 2c: Job demands are negatively related to LTPA *via* fatigue*	√	√	√
Research question 2			
* Hypothesis 3a: Job control is positively related to LTPA*	X	X	X
* Hypothesis 3b: WTC is positively related to LTPA*	X!	X!	X!
* Hypothesis 4a: Job control attenuates the negative association between job demands and fatigue*	X	X	X
* Hypothesis 4b: WTC attenuates the negative association between job demands and fatigue*	X	X	X
Research question 3			
* Hypothesis 5a: Autonomous motivation attenuates the negative association between fatigue and LTPA*	X!	X!	X!
* Hypothesis 5b: Spontaneous action planning attenuates the negative association between fatigue and LTPA*	X	X	X
Research question 4			
* Hypothesis 6: Job demands negatively relate to LTPA change*	X	X	X
* Hypothesis 7a: Fatigue negatively relates to LTPA change*	X	X	X
* Hypothesis 7b: Job demands negatively relate to LTPA change *via* fatigue*	X	X	X
* Hypothesis 8a: Job control positively relates to LTPA change*	X	X	X
* Hypothesis 8b: WTC positively relates to LTPA change the following year*	X	X	X
* Hypothesis 9a: Autonomous motivation attenuates the negative association between fatigue and LTPA change*	X!	X/X!	X!
* Hypothesis 9b: Spontaneous action planning attenuates the negative association between fatigue and LTPA change*	X	X	X

*Note.* √ = hypothesis confirmed; X = hypothesis rejected; X! = hypothesis rejected and opposite effect found

The ‘/’ is used to indicate different findings between analyses with job control as moderator (before dash) or

WTC as moderator (after dash)

^a^Robust 1 = robustness analyses in which cases with outliers (> 3SD) in LTPA have been removed. ^b^Robust 2 = robustness analyses in which the control variables are not included in the models anymore

## Discussion

In this study, we investigated the association between demanding (i.e., high workload and time pressure), sedentary work and participation in leisure–time physical activity (LTPA). We used a multidisciplinary approach to investigate factors within and outside the work environment that might explain or affect this relationship. Our results only provided support for a weak indirect association between job demands, fatigue and LTPA in our cross-sectional analyses. For all other hypotheses, no support or even opposing effects were found. Specifically, we could not confirm that job control or worktime control positively related to LTPA or attenuated a negative association between job demands and fatigue. Also, no evidence was found that autonomous motivation or spontaneous action planning buffered against a negative association between fatigue and LTPA. Finally, none of the expected associations were confirmed in our longitudinal analyses. Thus, within our sample of sedentary employees, we can only conclude that there is evidence for a weak and indirect association between work and LTPA.

It seems surprising that so many of our hypotheses were not confirmed. However, a closer look at our study sample provides an explanation. Our participants were relatively active employees with healthy working conditions, which was reflected in their relatively low levels of physical inactivity (only 15.1% was insufficiently active) and their relatively high levels of job control (i.e., only 1% reported to have no job control at all). It is very likely that this restricted range in our employee sample explains why we were unable to confirm the expected associations. For example, Fransson et al. (2012) report that the association between psychosocial work characteristics and LTPA is relatively weak and most pronounced for job control. Moreover, they showed that the odds of being sufficiently active decreased exponentially with decreasing job control. Finally, high levels of job control are thought to buffer against the negative consequences of high job demands for participation in LTPA (Häusser and Mojzisch 2017). Thus, it is plausible that within the present employee sample reporting high levels of job control, the expected associations between job demands, job control and LTPA were too weak to be detected or even nonexistent. While speculative, this provides indirect support for the prominent role of job control in the crossover effects from psychosocial work characteristics to LTPA.

In this light, it is interesting that even within our selective group of employees, we found a weak, negative indirect association between job demands and LTPA, via fatigue (hypothesis 2a–c). This finding is in line with previous experimental and diary studies showing negative (indirect) associations between job demands, fatigue and physical behavior (Brown and Bray 2019; Häusser et al. 2018; van Cutsem et al. 2017). However, our findings show that this association also applies to a large sample of employees working in relatively healthy conditions and while using state-like measures of both fatigue and job demands. Thus, in line with the long-held view that fatigue is characterized by an aversion to exert effort (Hockey 2011; Kanfer 2011; Thorndike 1914; van der Linden 2011), it seems that the subjective experience of fatigue, rather than demanding work itself, is an important factor linking sedentary work to participation in LTPA.

### Work-related moderators

We found no evidence for buffering effects of job control or WTC in the association between job demands and fatigue (hypothesis 4a and 4b). These findings contradict dominant job stress models which suggests that job control and WTC attenuate the negative impact of job demands on indicators of employee well-being, such as fatigue (Bakker and Demerouti 2007; Karasek 1979; Nijp et al. 2012). While the restricted range in our sample might explain this finding, previous research has also failed to provide consistent evidence for the stress-buffering effects of job control (for overviews, see Guthier et al. 2020; Häusser et al. 2010; van der Doef and Maes 1999) and researchers have tried to explain this before. One explanation holds that the buffering effects of job control are more likely when job demands and control are matched (Häusser et al. 2010). However, this matching principle cannot account for the current findings, as job demands and job control and WTC were deliberately matched in the present investigation (i.e., all focused on the time domain). Other explanations for the absence of moderating effects are that the power to detect such moderating effects is lower or that the effects do not exist (Taris 2006). However, as outlined by Häusser et al. (2010), these suggestions also do not seem to explain the absence of significant moderation effects of job control. As such, the most plausible explanation seems to be that we did not find buffering effects of job control and WTC because these effects depend upon individual differences and other job characteristics (for an overview, see Kubicek et al. 2017). For example, individuals might differ in their desire for control (or need for autonomy), which affects whether job control or WTC has a positive or negative impact on consequential well-being (e.g., fatigue; Parker et al. 2009; van Yperen et al. 2014). Similarly, job control and worktime control might be less beneficial for employees experiencing high time pressure and task complexity at work, due to the additional executive processes job control and WTC demand (e.g., planning and decision-making; Kubicek et al. 2017). Thus, it seems best to conclude that variation in unmeasured individual and work characteristics underlies the absence of buffering effects of job control and WTC within the present sample.

Interestingly, we found a weak *negative* association between WTC and LTPA (hypothesis 3b). In contrast to the proposed time regulation mechanism (Nijp et al. 2012), our results indicate that employees with more autonomy over their working times participate less in LTPA. While WTC provides a great opportunity for scheduling LTPA into a busy agenda, it is possible that the flexible working times hinder habit formation with respect to LTPA. To form a habit, an activity should repeatedly be performed within similar contextual cues (Wood 2017). If the timing of physical activity participation frequently changes due to varying worktimes, the likelihood to establish physical activity habits will be reduced (Wood et al. 2002). As habits are important predictors of physical activity (Rebar et al. 2016), the flexibility that WTC provides might actually backfire on participation in LTPA through reduced habit formation. However, more research will be needed to test and confirm these assumptions.

### Person-related moderators

Our results provide no support for the expectation that autonomous motivation or spontaneous action planning help fatigued employees to overcome their intolerance for effortful activities such as LTPA (Gollwitzer and Sheeran 2006; Werner et al. 2016; hypothesis 5a and 5b, respectively). With respect to autonomous motivation, our findings even showed that the negative association between fatigue and LTPA was stronger among employees with higher levels of autonomous motivation for LTPA. Close inspection of the LTPA scores suggest a floor effect among employees with low levels of autonomous motivation (i.e., standardized scores < − 1) which is represented by the high density of LTPA scores close to zero in this subgroup (see Supplemental Figure S1A). This floor effect is not present at high levels of autonomous motivation (i.e., standardized scores > 1; See Supplemental Figure S1B). Thus, it seems that the negative association between fatigue and LTPA is suppressed by the already low levels of LTPA among employees with low autonomous motivation and that the association between fatigue and LTPA becomes stronger at higher levels of autonomous motivation because this floor effect disappears. Together, these findings suggest that the adverse motivational consequences of fatigue are a robust barrier for participation in LTPA, especially affecting both employees with high autonomous motivation for physical activity and those with concrete action plans for participation in physical activity. This contradicts our expectation that autonomous motivation and spontaneous action planning buffer against the negative consequences of work-related fatigue for participation in LTPA. It could very well be that when individuals feel fatigued, the effortful nature of physical activities themselves is too aversive to overcome with strong autonomous motivation or concrete action plans. Alternative strategies, such as promoting low-effort physical activities (Ekkekakis et al. 2011), might therefore be more suitable to keep fatigued employees physically active.

### Longitudinal associations

Finally, with regard to our longitudinal investigation, we found no evidence for any of the expected longitudinal associations (hypotheses 6–9). Most associations were also not found cross-sectionally and have been discussed before. However, it should be noted that the significant indirect association between job demands, fatigue and LTPA on a cross-sectional level was not replicated longitudinally (hypotheses 6, 7a and 7b). This does not align with findings from de Vries et al. (2016), who found reciprocal associations between fatigue and LTPA change in the following year. An important distinction is that de Vries et al. (2016) measured fatigue using an exhaustion scale (Schaufeli and van Dierendonck 2000) while in the present study, fatigue was measured with a general work–fatigue scale (Michielsen et al. 2004). As the exhaustion scale measures fatigue of a higher severity than the currently employed fatigue scale, it seems plausible that only these severe levels of fatigue predict subsequent changes in LTPA. Thus, it seems that any directional effects of fatigue on changes in LTPA are nonexistent or too weak to be detected among employees with relatively healthy working conditions (i.e., high job control).

### Limitations and future directions

An important strength of the present study was that the association between work and LTPA was investigated from an interdisciplinary perspective, including insights from social–cognitive and occupational health psychology. As such, the role of several theoretically relevant but as yet overlooked constructs, such as WTC, autonomous motivation and spontaneous action planning, were investigated here. This is valuable for developing a comprehensive understanding of the association between work and participation in LTPA. Another asset of this study was that all investigated hypotheses were registered before data collection in an online repository. Preregistration strongly limits the likelihood to report effects that do not represent true population effects (i.e., false positives; Nosek and Lakens 2014; Simmons et al. 2011). As such, the present study provides an exemplary case of reproducible science within occupational health psychology.

At the same time, several limitations should be considered when interpreting the present findings. As discussed before, our sample was relatively well educated and only a few employees reported low levels of job control. Since levels of LTPA tend to be especially low among individuals with a low socioeconomic status (Beenackers et al. 2012) and with adverse levels of job control (Fransson et al. 2012) it will be valuable to investigate how our findings compare to those that would be obtained in a sample with lower levels of education and job control. This will provide important additional insight into the qualifying factors for an association between work and LTPA.

Another limitation is that our constructs of interest were only measured twice, with an one-year time lag in between. As such, we might have overlooked short-lived or nonlinear relationships between work characteristics and LTPA (Dormann and van de Ven 2014; Guthier et al. 2020). For example, it is possible that job demands have an impact on participation in LTPA, but only on a daily, weekly or monthly basis. Moreover, there might be curvilinear relationships, which can only be detected when including a third measurement moment. Therefore, it would be interesting to include additional measurement moments in future research to investigate alternative dynamic relationships between work and LTPA. Such additional measurements would complement our understanding of the association between work and LTPA by providing insight into the dynamic processes through which a potential crossover effect evolves over time.

Finally, LTPA was assessed using self-report. While this method was best suitable for our domain-specific assessment of physical activity in a large sample of employees, it has limited validity due to both over- and underestimation of physical activity levels (Prince et al. 2008). As such, it is possible that other patterns of relations emerge when LTPA is assessed directly using wearables such as heart-rate monitors or accelerometers. With the increasing availability of relatively low-cost and noninvasive wearable devices, measuring LTPA with direct measures will become more feasible and would complement our understanding of the association between work and LTPA.

### Implications and conclusion

The findings of this preregistered study have several theoretical and practical implications. Our study only provides support for a weak indirect association between job demands and LTPA at a cross-sectional level. This highlights the role of fatigue linking demanding sedentary work to LTPA. However, the fact that this indirect association was not observed in our longitudinal analyses suggests that while high job demands, fatigue and low levels of LTPA co-occur, there is no evidence for causality. The negative consequences of job demands for subsequent physical activity behavior in experimental and diary studies (Abdel Hadi et al. 2020; Brown and Bray 2019; Häusser et al. 2018) do not seem to apply to the currently investigated long-term associations between work characteristics and LTPA. On this time scale, it seems that previously reported associations between work and LTPA were reciprocal and mainly driven by rather severe forms of job control and fatigue (de Vries et al. 2016; Fransson et al. 2012). From a practical perspective, these findings show that redesigning work will need to be complemented by additional strategies in order to increase levels of LTPA. Work might affect LTPA to some extent in extreme cases, but clearly, other factors should be addressed as well. Our study thus provides a nuanced view on the association between demanding sedentary work and LTPA. In the absence of very low levels of job control, psychosocial work characteristics do not make a significant difference with respect to participation in LTPA.

### Electronic supplementary material

Below is the link to the electronic supplementary material.

Supplementary file1 (PDF 180 KB)

## Data Availability

All materials and analyses scripts are publicly available at the Open Science Framework (OSF) and can be accessed at https://osf.io/g7xb3/.

## Appendix

See Tables A1, A2 and A3.

**Table A1:** Participant exclusion per subsample

	Cross-sectional	Longitudinal (full panel)
	Frequency	Frequency
Age > 2-year increment	–	6
Age decrement	–	6
Work < 32 h	43	46
Work > 48 h	6	7
Work missing	43	51
**Total**	**92**	**116**

**Table A2:** Means, standard deviations and percentages of all moderator and control variables

Variable	Cross-sectional		Longitudinal (full panel)	
	Mean(SD)/%	Theoretical range	Mean(SD)/%	Theoretical range
Spontaneous action plans	2.68 (0.94)	1–4	2.73 (0.91)	1–4
Autonomous motivation	4.67 (1.46)	1–7	4.62 (1.50)	1–7
Controlled motivation	2.54 (0.95)	1–7	2.51 (0.96)	1–7
Amotivation	1.57 (0.94)	1–7	1.60 (0.98)	1–7
Alternative physical activities	98.25 (0.94)	0–∞	96.77 (74.01)	0–∞
Timing of physical activities	Externally determined = 9.4% Varying = 24.2% Self-determined = 66.4%	–	Externally determined = 9.3% Varying = 25.1% Self-determined = 65.6%	–
Composition of physical activities	Always alone = 45.3% Mostly together = 54.7%	–	Always alone = 45.9% Mostly together = 54.1%	–
Dog ownership	No = 85.6% Yes = 14.4%	–	No = 86.2% Yes = 13.8%	–

**Table A3:** Changes in pathway estimates for the robustness analysis excluding outliers in LTPA

Model	Changed pathway(s)	Change	*p* value
Model 1 (Job Control): cross	Demands → LTPA	n.s	0.085
Model 2 (WTC): cross	Total effect WTC → LTPA	n.s	0.126
Model 3 (Job Control): longi	Autonomous motivation T2 × Work fatigue T1 → LTPA change	n.s	0.057
	Action Planning T2 → LTPA change	Significant	0.009
Model 4 (WTC): longi	Action Planning T2 → LTPA change	Significant	0.000

*Note*. Only changes in significance or changes in direction are presented here

## Footnotes

1 Originally, we planned to perform step-wise structural equation modelling. Based on expert advice, we used path-analysis instead. This analysis is very similar and was better suited for testing our hypotheses.

2 We used the total approach (i.e., job demands, job control and their interaction as continuous predictors) to investigate the moderating effects of job control and WTC since it uses all available information and is therefore considered the gold standard (Courvoisier and Perneger 2010). For completeness, we preregistered to also apply the quadrant approach. Here, four job types are created based on median splits in job demands and job control scores and this new variable then serves as an unordered categorical predictor. However, it was impossible to apply this latter approach due to the distribution of control scores within our sample.

3 All possible pathways were estimated in order to perform the path analyses and test our hypotheses. The analyses scripts on the Open Science Framework (https://osf.io/g7xb3/) provide detailed overviews of all estimates.
