# Supplementary material for: Sedentary work and participation in leisure–time physical activity
Source: Int Arch Occup Environ Health. 2021 Sep 15;95(2):509–25. doi: 10.1007/s00420-021-01750-7 (PMC8795020; doi:10.1007/s00420-021-01750-7)
Supplement: Supplementary file 1 — Supplementary file1 (PDF 180 KB) [file 420_2021_1750_MOESM1_ESM.pdf]

Supplemental Materials

Table S1

Overview of Measured Constructs and Related Scales

Construct	Scale
Leisure-time physical activity	International Physical Activity Questionnaire, long version, last 7 days, leisure-time scale
Job demands	Job Content Questionnaire, demands scale
Job control	Job Content Questionnaire, control scale
Worktime control	Worktime Control Access subscale
Fatigue	Fatigue Assessment Scale
Motivation for physical activity (Autonomous, Controlled and Amotivation)	BREQ-4
Physical activities in non-leisure domains	SQUASH
Spontaneous action planning	-
Gender	-
Age	-
Highest level of completed education	-
Contractual working hours	-
BMI	-
Social composition of leisure-time physical activities	-
Control over timing of leisure-time physical activities (i.e., fixed or unfixed training moments)	-
Household composition	-
Hours worked overtime	-
Dog ownership	-

Note. See original manuscript for full references of the scales used.

Figure S1

Distribution of LTPA for Employees with Low (A) and High (B) Autonomous Motivation

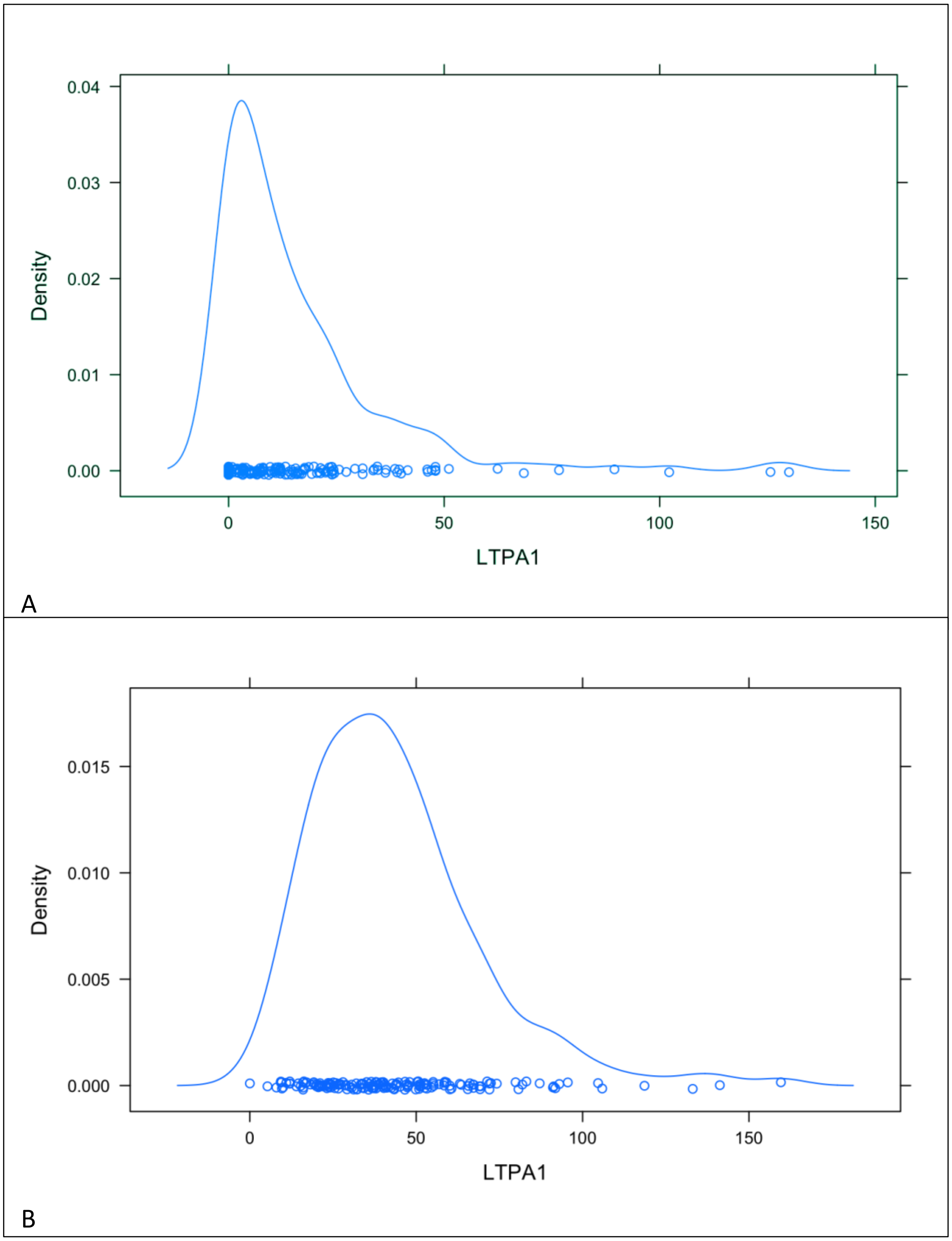

Note. Standardized autonomous motivation below -1 was ‘low’ (Panel A) and standardized autonomous motivation above 1 ‘high’ (Panel B).
